# Risk factors of bladder stones in neurogenic lower urinary tract dysfunction: A real‐world study

**DOI:** 10.1002/bco2.330

**Published:** 2024-02-01

**Authors:** Sandra Möhr, Saskia Fassbind, Brigitta Gahl, Hans‐Helge Seifert, Kathrin Bausch

**Affiliations:** ^1^ Department of Urology REHAB Basel Basel Switzerland; ^2^ University of Basel Basel Switzerland; ^3^ Department of Urology University Hospital of Basel Basel Switzerland; ^4^ Surgical Outcome Research Center Basel University Hospital of Basel Basel Switzerland

**Keywords:** calcium phosphate, risk factors, spinal cord injury, urinary tract infections, urolithiasis

## Abstract

**Objective:**

The objective of this study is to investigate the incidence and risk factors for stone formation and recurrence in patients with neurogenic lower urinary tract dysfunction (NLUTD) in a real‐world cohort.

**Materials and methods:**

A retrospective cohort study was conducted on all patients with NLUTD who underwent bladder stone treatment between 2010 and 2022. Univariate and multivariate Cox models were used to identify the potential risk factors for stone recurrence.

**Results:**

Among 114 patients included in the study, 30% experienced stone recurrence. The most common stone components were carbonate apatite phosphate and magnesium ammonium phosphate. The overall recurrence rate was 14 cases per 100 patient years. Neurogenic detrusor overactivity had the highest recurrence rate. Risk factors for stone recurrence in the multivariate analysis were intermittent and suprapubic catheterization, and recurrent urinary tract infection (rUTI).

**Conclusions:**

Patients experienced multiple bladder stone recurrences. Close monitoring of bladder pressure and UTI with restrictive catheter application may reduce the risk of stone recurrence.

## 1 INTRODUCTION

Bladder stones account for ~5% of all urinary stone cases in industrialized countries^1^ but are responsible for 8% of all urolithiasis‐related deaths.^2^ Stones usually form because of incomplete emptying of the bladder, which allows accumulation of lithogenic substances.^3^

Neurogenic lower urinary tract dysfunction (NLUTD) is a major risk factor for bladder stones.^4^ Owing to the underlying neurological diseases, patients experience more complex challenges in the diagnosis and treatment of urinary stones compared with the general population. In addition, bladder stones tend to recur more frequently and lead to increased morbidity in NLUTD patients.^5^ Therefore, patients with NLUTD should be monitored closely for bladder stones.

The incidence of bladder stones in NLUTD patients varies widely. In patients with spinal cord injury (SCI), the incidence has been reported to range from 3.3%^6^ to 36%.^4^ Several potential risk factors associated with NLUTD contribute to bladder stone formation, including bladder management techniques such as intermittent catheterization (CIC) or indwelling catheters,^7^ urinary diversion with the intestine^8^ and recurrent urinary tract infections (rUTI; >2 UTI in 6 months or 3 in 12 months), particularly those caused by urease‐forming bacteria.^9^ However, data regarding the relationship between metabolic anomalies, urine parameters and stone composition are conflicting.^7^, ^10^, ^11^

Limitations exist in previous studies that investigated urinary stones in patients with NLUTD. Several of these studies were relatively outdated,^4^, ^12^ included kidney stones,^7^ or were examined for only one underlying disease,^8^, ^13^ rather than providing insight into the real‐world NLUTD cohort typically observed by urologists. Consequently, the incidence and risk factors cannot often be determined, and apart from individual recommendations,^14^, ^15^ specific guidelines do not currently exist.^16^, ^17^

The present study was conducted to characterize bladder stones and stone recurrence in a real‐world NLUTD cohort and assess the potential risk factors for recurrent stone formation.

## 2 MATERIALS AND METHODS

### 2.1 Study design and setting

This retrospective cohort study was performed at the Department of Urology of REHAB Basel, Basel, Switzerland, a neurorehabilitation and paraplegiology centre.

The Ethics Committee of Northwestern and Central Switzerland approved the study protocol and waived the requirement for informed consent (no. 2022‐00365). This study was conducted in accordance with the ethical standards of the 1964 Declaration of Helsinki. We adhered to the Strengthening the Reporting of Observational Studies in Epidemiology (STROBE) guidelines when reporting study results.

### 2.2 Patient selection

All consecutive patients with NLUTD who received neuro‐urological care at REHAB were screened. Between 2010 and 2022, 4049 patients with NLUTD were seen in 10 650 consultations, of whom 114 (2.81%) had bladder stones and were eligible for inclusion in this study. Kidney stones were not a factor. The exclusion criteria were: missing outcome data (e.g. stone composition or urine results) or refusal to participate in any clinical study project involving the secondary use of routine healthcare data.

### 2.3 Data collection and definitions

Patients were identified from ongoing case statistics on stone analyses and inquiries at the laboratory where the stones were analysed. Stone clearance was checked endoscopically, and all stones were analysed. The stone size varied between 3 cm and a few millimetres. Urine composition and urine culture results were obtained. Blood parameters were not recorded. The entire cohort of patients with NLUTD was identified at the REHAB Basel. Two independent investigators (S.M. and S.F.) collected and reviewed all relevant data obtained from the in‐house electronic medical records or paper archives. The records were entered into a study database via electronic case report forms.

All the patients underwent regular examinations at our centre, including video‐urodynamics, endoscopy, laboratory tests and ultrasonography.^17^ Since 2010, an extended follow‐up regimen has been implemented as recommendations from Ord et al.^14^ and Ost and Lee^15^ for patients with permanent catheters. These patients underwent cystoscopy every 1–1.5 years if possible and annual sonography of the urinary tract. Patients without indwelling catheters underwent cystoscopy if clinically necessary, for example, in the case of macrohematuria or suspected stone formation. We only use silicone indwelling catheters, and they are routinely changed every 6 weeks in our patients with an uncomplicated urological course and earlier in case of clinical need. Radiography/CT was performed if stones were suspected in the upper urinary tract.

Our primary objective was to analyse bladder stones and stone recurrence in patients with NLUTD. The second objective was to identify potential risk factors for stone recurrence. At first stone occurrence, patient characteristics (e.g. age, sex, underlying disease, bladder function and bladder management) were assessed. Underlying diseases were categorized as spinal (congenital or acquired SCI), supraspinal (i.e. traumatic brain injury, cerebral palsy, wakeful coma and cerebral perfusion disorder) and multiple sclerosis (MS). We found no isolated peripheral neuropathies or Parkinson's disease. Nevertheless, the study population can be considered a real‐world cohort. UTIs were defined in accordance with European Association of Urology's (EAU) guidelines^17^ and diagnosed by urologists.

Routine clinical follow‐up was scheduled according to the treating physician's recommendations or when the patient had physical complaints; however, all patients were followed. The same data were collected for all the recurrences.

### 2.4 Statistical analysis

Continuous variables are presented as mean with standard deviation (SD) if normally distributed, or as median with interquartile range (IQR) if skewed. Categorical variables are presented as numbers with percentages with comparisons based on Fisher's exact test, or as non‐parametric trends, if explicitly mentioned.

Regarding the primary aim of the study, stone composition was described as the number of occurrences of specific components and cross‐tabulated with patient and disease characteristics.

To investigate the risk of stone recurrence, event rates were calculated with 95% confidence intervals (CIs) for the entire study cohort, underlying disease and bladder dysfunction type. Univariate Cox regression analyses were conducted with multiple events and hazard ratios (HRs) determined for age, sex, underlying disease, bladder function, bladder management, rUTI and median urine pH values 3 months preoperatively, which were modelled as time‐varying covariates. Furthermore, underlying disease, bladder function, bladder management, rUTI and median urine pH values 3 months preoperatively were included as covariates in the multivariable Cox regression analysis. To reduce bias and avoid loss of statistical power, multiple imputations were conducted using chained equations, adding 100 imputations for each missing value. Statistical significance was set at *p* < 0.05.

All analyses were performed using Stata 16.0 (StataCorp LLC, College Station, TX, USA). An overview plot was created using R (The R Project for Statistical Computing, https://www.r-project.org, version 4.1).

## 3 RESULTS

### 3.1 Basic characteristics

The study included 114 patients with 206 stone episodes and 161 stone composition analyses.

The mean patient age at the time of the first stone surgery was 54 years (SD, 16 years), and 48 patients were female. The interval between the onset of underlying neurological disease and first stone ranged from 32 days to 59.1 years (median 9.2 years), and the majority of first stones occurred >1 year after disease onset.

An underlying spinal disease was observed in 49% of the patients (*n* = 56). Furthermore, none of the following comorbidities were present: gout, hyperparathyroidism or chronic inflammatory bowel disease.

Patients tended to be severely affected neurologically and limited in their ability to work. The median functional independence measure (FIM) assessment score was 48, IQR 30 to 75, and ranged from 19 to 126 points.

After the first stone event, recurrences occurred in 30% (*n* = 34) with an average of 0.8 stones recurring in all patients. A single stone recurrence occurred in 15% of patients; another 15% experienced more than one stone recurrence (up to 9; Table 1A; Figure S1). Among the stones, 19% had only one component. The most frequent stone component was carbonate apatite phosphate, present in 88% of stones. A summary of the stone composition is presented in Table 1B.

**TABLE 1:** (A) Patient characteristics and (B) stone characteristics showing stone composition and patient characteristics per included stone.

(A) Patient characteristics	Total (*N* = 114)
Age at first stone surgery in years, mean (SD)	54 (16)
Gender, *n* (%)	
Female	48 (42)
Male	66 (58)
Latency between onset of underlying disease and first stone surgery, *n* (%)	
≤1 year	32 (28)
>1 year	82 (72)
Number of underlying neurological disease per patient, *n* (%)	
1	102 (89)
2	11 (10)
3	1 (0.88)
Main underlying disease, *n* (%)	
Spinal^a^	56 (49)
Supraspinal^b^	32 (28)
Multiple sclerosis	26 (23)
Urine pH 3 month preoperatively, mean (SD)	7.0 (1.1)
Recurrent UTI (≥3 per year), *n* (%)	37 (32)
Stone recurrences, *n* (%)	
0	80 (70)
1	17 (15)
>1	17 (15)

(B) Stone characteristics	
Stone composition, *n* (%)	Total (*N* = 161)
Calcium oxalate monodydrate (whewellite)	43 (27)
Calcium oxalate dihydrate (weddelite)	32 (20)
Carbonate apatite phosphate (dahllite)	141 (88)
Calcium hydrogen phosphate dihydrate (brushit)	6 (3.7)
Uric acid (uricite)	10 (6.2)
Uric acid dihydrate	4 (2.5)
Ammonium urate	9 (5.6)
Magnesium ammonium phosphate (struvit)	67 (42)
Others	1 (0.6)
One component only	31 (19)
Stone composition grouped per *n* of stones	
Calcium oxalate (whewellite, weddelite)	66 (41)
Calcium phosphate (dahllite, brushit)	144 (89.4)
Urate (uric acid, uric acid dihydrate, ammonium urate)	16 (9.9)

Patient characteristics per stone	Total (*N* = 206)
Bladder function, *n* (%)	
NDO and DSD	178(86)
NDU	13 (6.3)
Unknown	15 (7.3)
Bladder management, *n* (%)	
Spontaneous micturition	13(6.3)
Intermittent catheterization	48 (23)
Transurethral catheterization	36 (17)
Suprapubic catheterization	101 (49)
Urinary condom	7 (3.4)
Pads	6 (2.9)
Urinary diversion by ileum conduit	0 (0)
Urinary diversion by bladder augmentation or neobladder	3 (1.5)

Abbreviations: DSD, detrusor sphincter dyssynergia; *N*, total number; *n*, number; NDO, neurogenic detrusor overactivity; NDU, neurogenic detrusor underactivity; SD, standard deviation; UTI, urinary tract infection.

^a^ Spinal representing spinal cord injury;

^b^ supraspinal including traumatic brain injury, cerebral palsy, wake coma and cerebral perfusion disorder.

Neurogenic detrusor overactivity (NDO) with detrusor sphincter dyssynergia (DSD) was the most common bladder dysfunction (86%), and the main method of bladder management was a suprapubic catheter (SPC; 49%; Table 1B).

### 3.2 Stone recurrence

Figure S1 shows stone recurrences during the observation period and the underlying diseases. The overall recurrence rate was 14 per 100 patient years and was similar among the underlying diseases. The highest recurrence rate was observed in patients with NDO and DSD, at 17 per 100 patient years (Table 2A).

**TABLE 2:** (A) First and any recurrence in relation to underlying disease and bladder function. (B) Stone recurrence in relation to bladder management, stone composition and recurrent urinary tract infections.

Potential risk factor/variable	First recurrence			Any recurrence		
	*n* of events	Patient years	Rate per 100 patient years	*n* of events	Patient years	Rate per 100 patient years
All patients	34	465	7 (5–10)	92	664	14 (11–17)
Underlying disease						
Spinal	14	260	5 (3–9)	44	345	13 (9–17)
Supraspinal	11	103	11 (6–19)	23	156	15 (10–22)
Multiple sclerosis	9	102	9 (5–17)	25	162	15 (10–23)
Bladder function						
NDO	29	342	8 (6–12)	87	512	17 (14–21)
NDU	2	54	4 (1–15)	2	56	4 (1–14)
Unknown	3	69	4 (1–13)	3	96	3 (1–10)

Potential risk factor/variable	No recurrence (N = 80)	1 Episode (N = 34)	>1 Episode (N = 92)	*p*	p*
Bladder management					
Spontaneous micturition	7 (8.8%)	6 (18%)	0 (0%)	<0.001	0.015
Intermittent catheterization	19 (24%)	4 (12%)	25 (27%)	0.19	0.564
Transurethral catheterization	18 (23%)	2 (5.9%)	16 (17%)	0.09	0.411
Suprapubic catheterization	31 (39%)	20 (59%)	50 (54%)	0.06	0.046
Urinary condom	7 (8.8%)	0 (0%)	0 (0%)	0.003	0.002
Pads	2 (2.5%)	2 (5.9%)	1 (1.1%)	0.25	0.522
Urinary diversion by ileum conduit	0 (0%)	0 (0%)	0 (0%)		
Urinary diversion by bladder augmentation or neobladder	1 (1.3%)	2 (5.9%)	0 (0%)	0.035	0.455
Stone composition					
Calcium oxalate (whewellit and weddelite)	36 (47%)	14 (54%)	16 (27%)	0.020	0.022
Calcium phosphate (dahllite and brushit)	67 (88%)	18 (69%)	59 (100%)	<0.001	0.043
Urate (uric acid, uric acid dihydrate and ammonium urate)	6 (7.9%)	6 (23%)	4 (6.8%)	0.06	0.929
Magnesium ammonium phosphate (struvit)	25 (33%)	8 (31%)	34 (58%)	0.008	0.005
Recurrent UTI (≥3 per year)	22 (28%)	7 (21%)	49 (53%)	<0.001	

*Note*: p* refers to non‐linear trend.

Abbreviations: *N*, total number; NDO, neurogenic detrusor overactivity; NDU, neurogenic detrusor underactivity; UTI, urinary tract infection.

Stone recurrence tended to increase in SPC patients (Table 2B). Both calcium phosphate and magnesium ammonium phosphate stones recurred more than once (Table 2B). Table S1 shows the stone composition per episode and proportion of stone components relative to the first episode.

Patients with rUTI were more likely to experience >1 episode of recurrence (Table 2B). The underlying uropathogens are listed in Table S2. In most cases, these were either obligate or facultative urease‐forming bacteria (Table S2). The presence of magnesium ammonium phosphate stones was significantly associated with rUTI. The association between carbonate apatite phosphate stones and rUTI was not statistically significant (Table S3).

### 3.3 Risk factors for stone recurrence

In the univariate analysis, a higher mean urine pH 3 months preoperatively significantly increased the risk of stone recurrence, with an HR of 1.23 by unit increase. rUTI was associated with a double risk of stone recurrence. After adjusting for underlying disease, urine pH and rUTI, multivariate analysis showed that patients with both CIC and SPC had a triple risk of stone recurrence. After adjustment, the rUTI nearly doubled the risk of recurrence (Table 3).

**TABLE 3:** Potential risk factors for stone recurrence. Univariable and multivariable Cox regression, derived hazard ratios and *p*‐values.

Potential risk factor/variable	HR (95% CI)	*p*‐Value
Univariable		
Age in years	0.99 (0.97 to 1.01)	0.304
Gender, female	1.17 (0.64 to 2.16)	0.611
Underlying disease		
Spinal	Reference	
Supraspinal	0.98 (0.46 to 2.05)	0.949
Multiple sclerosis	1.12 (0.55 to 2.30)	0.759
Bladder function, NDU	0.27 (0.07 to 1.03)	0.055
Bladder management		
Intermittent catheterization	1.06 (0.54 to 2.08)	0.875
Transurethral catheterization	0.76 (0.27 to 2.12)	0.595
Suprapubic catheterization	1.49 (0.80 to 2.76)	0.210
Median urine pH 3 month preoperatively	1.23 (1.00 to 1.52)	0.048
Recurrent UTI, ≥3 per year	1.91 (1.10 to 3.32)	0.021
Multivariable		
Underlying disease		
Spinal	Reference	
Supraspinal	0.97 (0.42 to 2.21)	0.942
Multiple sclerosis	1.01 (0.42 to 2.42)	0.984
Bladder function, NDU	0.40 (0.10 to 1.68)	0.213
Bladder management		
Intermittent catheterization	2.76 (1.11 to 6.83)	0.028
Transurethral catheterization	1.98 (0.55 to 7.11)	0.293
Suprapubic catheterization	2.70 (1.23 to 5.92)	0.013
Urine pH 3 month preoperatively	1.22 (0.95 to 1.57)	0.116
Recurrent UTI, ≥3 per year	1.79 (1.04 to 3.07)	0.035

Abbreviations: CI, confidence interval; HR, hazard ratio; NDU, neurogenic detrusor underactivity; UTI, urinary tract infection.

## 4 DISCUSSION

The results of this study revealed frequent stone recurrences in patients with NLUTD in a real‐world setting. Therefore, close and long‐term monitoring should be performed in at‐risk patients (e.g. NDO, catheterization and rUTI).

To the best of our knowledge, this is the largest real‐world NLUTD bladder stone cohort study to date, in which an extended stone analysis was performed. The limitations of previous studies were the lack of distinction between the bladder and upper urinary tract, date of recurrence and stone analysis, and inclusion of only one neurological disease, without considering the entire NLUTD spectrum.^6^, ^13^, ^14^

The real‐world cohort in the present study consisted of 49% of patients with acquired or congenital SCI, 28% with various brain injuries up to wakeful coma and 23% with MS, representing a broad NLUTD spectrum. This cohort composition allows for the assessment of neurological diseases as risk factors for stone formation.

Most bladder stones in the NLUTD cohort were diagnosed at a later stage (>1 year after the neurological onset). In recent studies on patients with SCI, bladder stones were more likely to occur in the chronic phase than in the acute and post‐acute phases.^4^, ^13^ Therefore, long‐term monitoring of patients with NLUTD is warranted.

The chemical composition of the stones in the NLUTD cohort differed significantly from that in the general population, indicating that other factors may have caused stone formation. Although 19% of NLUTD stones in our study consisted of only one component, this rate was much higher in the general population (81%).^16^ Calcium oxalate stones were observed more frequently in the general population (41% vs. 70.4%). Carbonate apatite phosphate stones (dahllite) were more common in NLUTD patients (88% vs. 4.8%).^18^

In previous NLUTD studies, stones were either not completely analysed^4^, ^12^, ^13^ or information was not provided regarding mixed stones,^12^ and only infectious and non‐infectious stones^7^ or apatite and infectious stones were distinguished.^6^ Differentiating between partially infectious (carbonate apatite phosphate, i.e. dahllite) and non‐infectious (calcium hydrogen phosphate dihydrate, i.e. brushite) calcium phosphate (i.e. apatite) stones is necessary.^19^, ^20^ In the present study, dahllite and struvite were found in 88% and 42% of stones, respectively. A high prevalence (98%^12^ to 100%^6^) of infectious stones has been reported in paraplegic patients. Furthermore, when comparing bladder stones between MS and non‐MS patients, significantly more infectious stones were detected in patients with neurological disease.^7^ In the present study, infectious stones were found to contribute significantly to stone recurrence. These findings indicate that rUTI plays a significant role in stone formation and recurrence in patients with NLUTD.

The overall risk of urinary stone recurrence in the general population is high (~50%).^21^, ^22^ However, for bladder stones, data on the recurrence rates in both the general population and patients with NLUTD are scarce. Owing to the heterogeneity of the NLUTD cohort, most studies have focussed on urinary stones in general,^13^, ^15^ specific diseases such as SCI,^6^, ^14^ or only certain subgroups (e.g. women with indwelling catheters).^23^ Therefore, previously described recurrence rates varied between 16%^14^ and 23%.^6^

One third of the study cohort had recurrent stones, of which 50% had >1 recurrence. This high rate can be explained by the large number of catheterized patients, rUTI and stringent follow‐up. Owing to rigorous screening, some stones were detected before they became symptomatic.

When recurrence rates were analysed in specific subgroups, similar data were observed in groups of patients with spinal, supraspinal and MS diseases, supporting the decision to study the NLUTD cohort as a whole. Recurrence has been shown to be associated with bladder (dys‐)function rather than with the underlying disease. The highest recurrence rates were observed in patients with NDO and DSD. One reason for the association between stone recurrence and NDO and DSD could be the high intravesical pressure that results in hypoperfusion of the urothelium, which is prone to bacterial infection.^24^ rUTI was detected in 44% and 17% of the patients with NDO and NDU, respectively. Furthermore, DSD can cause a post‐void residual volume and increase the risk of rUTI.^25^ More than one third (37%) of the study cohort had rUTI. In the literature, this rate has been reported to be slightly higher during the first rehabilitation of SCI patients (43%).^26^ In the present study, rUTI led to >1 stone recurrence, and rUTI was a risk factor for stone recurrence in multivariate analysis. Infectious stones were the most common stones in patients with NLUTD.

Spontaneous micturition and incontinence were associated with the lowest risks of recurrence. SPC and CIC showed more recurrences and almost tripled the risk of recurrence. These findings are in agreement with those of a previous study that included patients with SCI, indicating a higher risk of stones with CIC and permanent catheters.^13^

However, in an earlier SCI study (1985–1990),^14^ an increased number of stones with transurethral catheters and SPC was observed; still, recurrence did not increase compared with CIC. Regarding the risk of stone recurrence in SCI patients, Bartel et al.^6^ reported the highest recurrence rates in patients with transurethral catheters, followed by SPC and CIC, with no increased risk of reflex emptying with the use of urinary condoms. Therefore, CIC is a reliable risk factor for recurrent stones because of the significantly increased rate of rUTI.^26^

The heterogeneous results for different modes of bladder management in the literature may be explained by the lack of assessment of bladder pressure reduction. The role of a potentially inadequate or unattenuated high‐pressure bladder when supplied with an indwelling catheter versus the mandatory attenuated bladder when using CIC cannot be assessed. However, because spontaneous and reflex emptying into the urinary condom has the lowest recurrence rate, the catheter itself may also be a risk factor. Incontinence tends to have fewer recurrences with the use of urinary condoms than with absorbent materials, which may be due to lower infection rates and the need to reduce incontinence pressure before reflex emptying into the urinary condom.^27^, ^28^

The present study has several limitations. First, the study was conducted at a single centre. Second, the sample size may not have allowed the detection of small effects. Third, this study may be limited by its retrospective design, and prospective multicentre investigations are needed to integrate this premise into current clinical practice. Fourth, the risk factors studied may not be completely independent of each other. Therefore, a multivariate analysis with appropriate correction was performed. Fifth, several parameters, particularly bladder function, are not static and may be affected by medication or bladder management. The specific influence of these parameters should be studied prospectively. Sixth, not all potential risk factors could be assessed, such as, for example, the hydration status of the patients or residual urine in patients without indwelling catheter.

## 5 CONCLUSION

Patients with NLUTD are at high risk of stone formation. The risk of recurrence is particularly high in rUTI (almost doubled), SPC and CIC (tripled), but also NDO with DSD and evidence of infection‐associated stones pose significant risks. These patients may require lifelong close monitoring and preventive measures to reduce their risk of stone formation, as mentioned in the Section 2: regular control cystoscopies for patients with indwelling catheters, in our centre every 12–18 months if feasible. Close control of bladder pressure and UTI with cautious application of catheters could help reduce the risk.

## AUTHOR CONTRIBUTIONS

Sandra Möhr contributed to protocol/project development, methodology, data collection, data analysis, manuscript writing and manuscript editing. Saskia Fassbind contributed to data collection. Brigitta Gahl contributed to data analysis and manuscript editing. Hans‐Helge Seifert contributed to protocol/project development and manuscript editing. Kathrin Bausch contributed to protocol/project development, methodology, data analysis, manuscript writing and manuscript editing.

Collaborators: Anais Schlenker‐Henssler^c^ (anais.schlenker@usb.ch) contributed to technical editing of the manuscript.

## CONFLICT OF INTEREST STATEMENT

All authors certify that they have no financial or personal relationships with any person or organization that could inappropriately influence their work. The authors declare that they have no conflicts of interest.

## Supporting information


**Figure S1.** Stone recurrence during observation time per patient and underlying disease.
**Table S1.** Stone composition per stone episode and correlation of stone composition at different stone episode to first stone episode.
**Table S2.** Uropathogens in relation to recurrence. *obligate urease‐forming bacteria; **facultative urease‐forming bacteria.
**Table S3.** Relation between stone composition and recurrent urinary tract infections.

## References

[1] Schwartz BF , Stoller ML . Nonsurgical management of infection‐related renal calculi. Urol Clin North am. 1999;26(4):765–778, viii. 10.1016/S0094-0143(05)70217-2 10584617

[2] Kum F , Mahmalji W , Hale J , Thomas K , Bultitude M , Glass J . Do stones still kill? An analysis of death from stone disease 1999‐2013 in England and Wales. BJU Int. 2016;118(1):140–144. 10.1111/bju.13409 26765522

[3] Merritt JL . Residual urine volume: correlate of urinary tract infection in patients with spinal cord injury. Phys Med Rehabil. 1981;62(11):558–561. 7316711

[4] DeVivo MJ , Fine PR , Cutter GR , Maetz HM . The risk of bladder calculi in patients with spinal cord injuries. Intern Med. 1985;145(3):428–430. 10.1001/archinte.145.3.428 3977510

[5] Kasabwala K , Borofsky M , Grove S , Lenherr SM , Myers JB , Stoffel JT , et al. Association of stone surgery with patient‐reported complications after spinal cord injury. Neurourol Urody. 2022;41(3):820–829. 10.1002/nau.24887 35114016

[6] Bartel P , Krebs J , Wöllner J , Göcking K , Pannek J . Bladder stones in patients with spinal cord injury: a long‐term study. Spinal Cord. 2014;52(4):295–297. 10.1038/sc.2014.1 24469146

[7] Ganesan V , Chen WM , Jain R , De S , Monga M . Multiple sclerosis and nephrolithiasis: a matched‐case comparative study. BJU Int. 2017;119(6):919–925. 10.1111/bju.13820 28220601

[8] Veenboer PW , Ruud Bosch JLH , van Asbeck FWA , de Kort LM . Urolithiasis in adult spina bifida patients: study in 260 patients and discussion of the literature. Int Urol Nephro. 2013;45(3):695–702. 10.1007/s11255-013-0445-8 23604705

[9] Bichler K‐H , Eipper E , Naber K . Infection‐induced urinary stones. UrologeA. 2003;42(1):47–55. 10.1007/s00120-002-0272-5 12574884

[10] Childs MA , Mynderse LA , Rangel LJ , Wilson TM , Lingeman JE , Krambeck AE . Pathogenesis of bladder calculi in the presence of urinary stasis. J Urol. 2013;189(4):1347–1351. 10.1016/j.juro.2012.11.079 23159588 PMC3777386

[11] Nevo A , Moore JP , Humphreys MR , Salih S , Keddis MA , Rose KM , et al. Does bladder stone composition predict kidney stone composition? Can J Urol. 2020;27(6):10450–10455. 33325347

[12] Burr RG . Urinary calculi composition in patients with spinal cord lesions. Arch Phys Med Rehabil. 1978;59(2):84–88. 623518

[13] Chen Y , DeVivo MJ , Lloyd LK . Bladder stone incidence in persons with spinal cord injury: determinants and trends, 1973‐1996. Urology. 2001;58(5):665–670. 10.1016/S0090-4295(01)01374-7 11711333

[14] Ord J , Lunn D , Reynard J . Bladder management and risk of bladder stone formation in spinal cord injured patients. JUrol. 2003;170(5):1734–1737. 10.1097/01.ju.0000091780.59573.fa 14532765

[15] Ost MC , Lee BR . Urolithiasis in patients with spinal cord injuries: risk factors, management, and outcomes. Curr Opin Urol. 2006;16(2):93–99. 10.1097/01.mou.0000193376.07071.ac 16479211

[16] Skolarikos A . EAU Guidelines on Urolithiasis 2022. Arnhem, 2022.

[17] Blok B (Chair). EAU Guidelines Neuro Urology. https://uroweb.org/guidelines/neuro-urology. 1 May 2023.

[18] Knoll T , Bach T , Humke U , Neisius A , Stein R , Schönthaler M , et al. S2k guidelines on diagnostics, therapy and metaphylaxis of urolithiasis (AWMF 043/025) compendium. UrologeA. 2016;55(7):904–922. 10.1007/s00120-016-0133-2 27325405

[19] Skolarikos A , Straub M , Knoll T , Sarica K , Seitz C , Petřík A , et al. Metabolic evaluation and recurrence prevention for urinary stone patients: EAU guidelines. Eur Urol. 2015;67(4):750–763. 10.1016/j.eururo.2014.10.029 25454613

[20] Siener R , Netzer L , Hesse A . Determinants of brushite stone formation: a case‐control study. PLoS ONE. 2013;8(11):e78996. 10.1371/journal.pone.0078996 24265740 PMC3827110

[21] Hesse A , Brändle E , Wilbert D , Köhrmann K‐U , Alken P . Study on the prevalence and incidence of urolithiasis in Germany comparing the years 1979 vs. 2000. Eur Urol. 2003;44(6):709–713. 10.1016/S0302-2838(03)00415-9 14644124

[22] Strohmaier WL . Course of calcium stone disease without treatment. What can we expect? Eur Urol. 2000;37(3):339–344. 10.1159/000052367 10720863

[23] Trop CS , Bennett CJ . Complications from long‐term indwelling Foley catheters in female patients with neurogenic bladders. Semin Urol. 1992;10(2):115–120. 1636070

[24] Vasudeva P , Madersbacher H . Factors implicated in pathogenesis of urinary tract infections in neurogenic bladders: some revered, few forgotten, others ignored. Neurol Urodyn. 2014;33(1):95–100. 10.1002/nau.22378 23460489

[25] Yan T , Liu C , Li Y , Xiao W , Li Y , Wang S . Prevalence and predictive factors of urinary tract infection among patients with stroke: a meta‐analysis. Am J Infect Contro. 2018;46(4):402–409. 10.1016/j.ajic.2017.10.001 29153643

[26] Anderson CE , Chamberlain JD , Jordan X , Kessler TM , Luca E , Möhr S , et al. Bladder emptying method is the primary determinant of urinary tract infections in patients with spinal cord injury: results from a prospective rehabilitation cohort study. BJU Int. 2019;123(2):342–352. 10.1111/bju.14514 30113757

[27] Yıldız N , Akkoç Y , Erhan B , et al. Neurogenic bladder in patients with traumatic spinal cord injury: treatment and follow‐up. Spinal Cord. 2014;52(6):462–467. 10.1038/sc.2014.41 24732167

[28] Fahimzad A , Taherian M , Dalirani R , Shamshiri A . Diaper type as a risk factor in urinary tract infection of children. Am J Infect Contro. 2010;20(1):97–100. PMC3446007 23056689

