# Supplementary material for: Risk factors of bladder stones in neurogenic lower urinary tract dysfunction: A real‐world study
Source: BJUI Compass. 2024 Feb 1;5(3):359–65. doi: 10.1002/bco2.330 (PMC10927923; doi:10.1002/bco2.330)
Supplement: Supplementary file 1 — Figure S1. Stone recurrence during observation time per patient and underlying disease. Table S1. Stone composition per stone episode and correlation of stone composition at different stone episode to first stone episode. Table S2. Uropathogens in relation to recurrence. *obligate urease‐forming bacteria; **facultative urease‐forming bacteria. Table S3. Relation between stone composition and recurrent urinary tract infections. [file BCO2-5-359-s001.pdf]

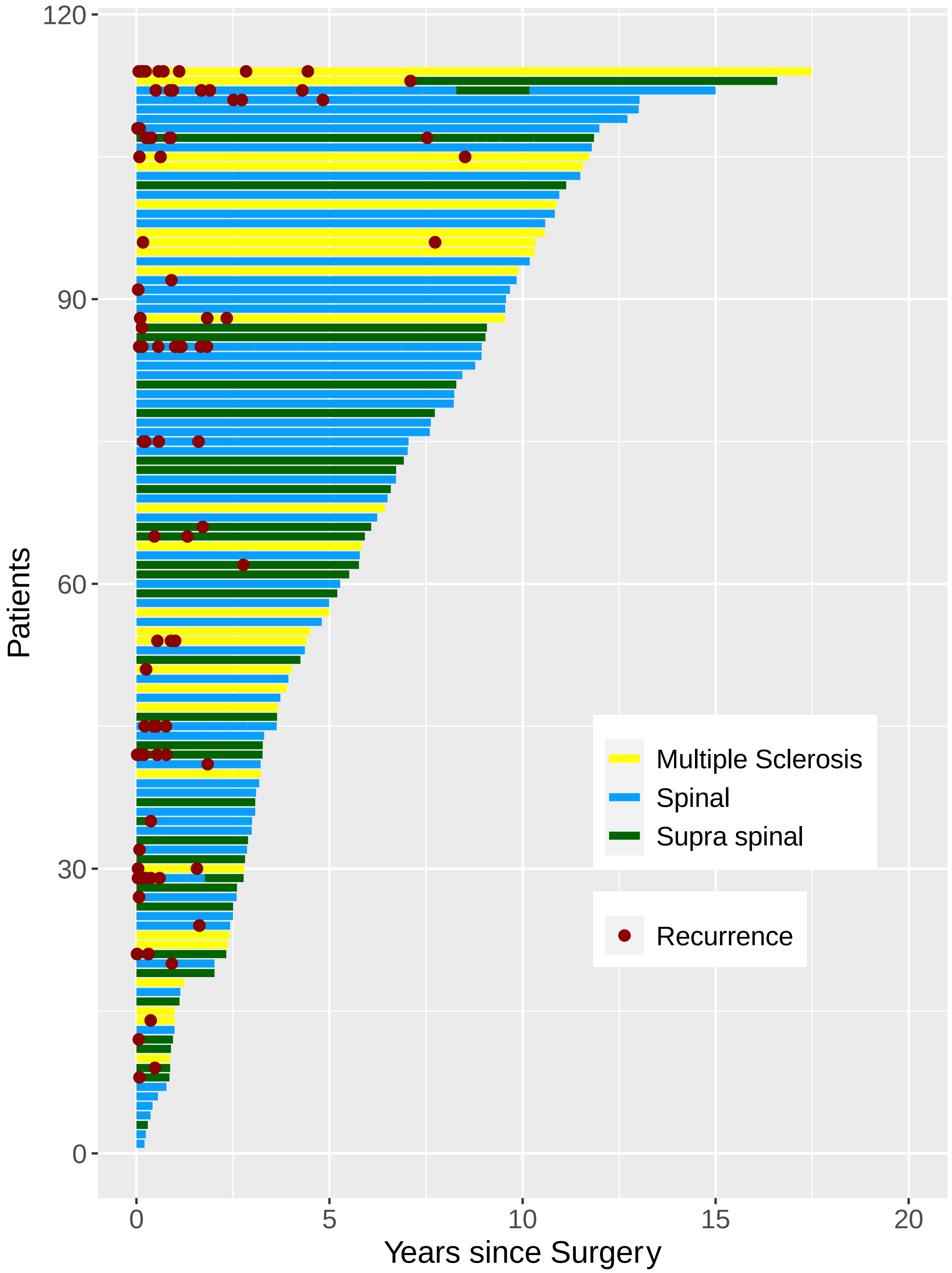

Figure S1: Stone recurrence during observation time per patient and underlying disease.

stone composition	stone episode					correlation of stone composition at stone episodes			
	1st	2nd	3rd	4th	5th	1st&2nd	1st&3rd	1st&4th	1st&5th
	48	11	3	3	2	3 (27%)	2 (67%)	2 (67%)	1 (50%)
Calcium oxalate monohydrate (Whewellite)	29	8	3	3	0	4 (50%)	1 (33%)	0 (0%)	0 (0%)
Calciumoxalate dihydrate (Weddelite)	22	4	3	1	0	1 (25%)	1 (33%)	0 (0%)	0 (0%)
Carbonate apatite phosphate (Dahllite)	81	25	12	8	6	15 (60%)	6 (50%)	4 (50%)	5 (50%)
Calcium hydrogen phosphate dihydrate (Brushit)	6	5	0	0	1	0 (0%)	0 (0%)	0 (0%)	0 (0%)
Uric acid (Uricit)	7	3	0	0	0	3 (100%)	0 (0%)	0 (0%)	0 (0%)
Uric acid dihydrate	2	2	0	0	0	1 (50%)	0 (0%)	0 (0%)	0 (0%)
Ammonium urate	5	0	1	2	1	0 (0%)	0 (0%)	0 (0%)	0 (0%)
Magnesium ammonium phosphate (Struvit)	35	12	7	4	3	9 (75%)	3 (43%)	1 (25%)	1 (33%)

Table S1: Stone composition per stone episode and correlation of stone composition at different stone episode to first stone episode.

	No Recurrence (N = 80)	One episode (N = 34)	>1 episode (N = 92)	p
Escherichia coli **	27 (34%)	10 (29%)	22 (24%)	0.37
Proteus species *	20 (25%)	5 (15%)	22 (24%)	0.50
Klebsiella species **	17 (21%)	6 (18%)	6 (6.5%)	0.014
Citrobacter species	5 (6.3%)	1 (2.9%)	14 (15%)	0.06
Pseudomonas aeruginosa **	20 (25%)	7 (21%)	17 (18%)	0.59
Enterobacter species	5 (6.3%)	2 (5.9%)	6 (6.5%)	1.00
Enterococcus species	15 (19%)	3 (8.8%)	18 (20%)	0.38
Staphylococcus aureus **	7 (8.8%)	2 (5.9%)	4 (4.3%)	0.47
coagulase negative Staphylococ. **	3 (3.8%)	0 (0.0%)	0 (0.0%)	0.13
Streptococci viridans	1 (1.3%)	0 (0.0%)	1 (1.1%)	1.00
Streptococci species	1 (1.3%)	0 (0.0%)	3 (3.3%)	0.66
Morganella morganii *	2 (2.5%)	2 (5.9%)	9 (10%)	0.14
Aerococcus urinae	1 (1.3%)	1 (2.9%)	2 (2.2%)	0.82
Corynebacterium ureal. *	0 (0.0%)	1 (2.9%)	3 (3.3%)	0.28
Achromobacter xylosans	0 (0.0%)	1 (2.9%)	0 (0.0%)	0.17
Candida albicans	1 (1.3%)	1 (2.9%)	2 (2.2%)	0.82
Acinetobacter spec	2 (2.5%)	2 (5.9%)	0 (0.0%)	0.06
Stenotrophomonas maltophilia	0 (0.0%)	0 (0.0%)	1 (1.1%)	1.00
Serratia marcescens **	2 (2.5%)	0 (0.0%)	3 (3.3%)	0.85
Haemophilus parainfluenzae	0 (0.0%)	1 (2.9%)	0 (0.0%)	0.17
Providentia rettgeri *	3 (3.8%)	0 (0.0%)	3 (3.3%)	0.74
Non-fermenting	0 (0.0%)	0 (0.0%)	1 (1.1%)	1.00
Lactobacillus	1 (1.3%)	1 (2.9%)	1 (1.1%)	0.56
sterile urine culture	15 (19%)	5 (15%)	8 (8.7%)	0.15

Table S2: Uropathogens in relation to recurrence. * obligate urease-forming bacteria; ** facultative urease-forming bacteria

N, total number

	no recurrent UTI (<3/year) (N = 93)	recurrent UTI(≥3/year) (N = 65)	p

Carbonate apatite phosphate (Dahllite)	78 (84%)	61 (94%)	0.08
Ammonium urate	5 (5.4%)	37 (57%)	1.0
Magnesium ammonium phosphate (Struvit)	30 (32%)	37 (57%)	0.003

Table S3: Relation between stone composition and recurrent urinary tract infections

UTI, urinary tract infection
